# Breathing across ages: a systematic review on challenges and components of transitional care for young people with asthma

**DOI:** 10.3389/fped.2024.1348963

**Published:** 2024-02-21

**Authors:** Luna Antonino, Kim Van Hoorenbeeck, Josefien van Olmen, Yaël Vanharen, Natwarin Janssens, Stijn Verhulst, Eva Goossens

**Affiliations:** ^1^ Centre for Research and Innovation in Care (CRIC), Department of Nursing Science and Midwifery, Faculty of Medicine and Health Sciences, University of Antwerp, Antwerp, Belgium; ^2^ Laboratory of Experimental Medicine and Pediatrics (LEMP), Department of Pediatrics, Faculty of Medicine and Health Sciences, University of Antwerp, Antwerp, Belgium; ^3^ Department of Pediatric Pulmonology, Antwerp University Hospital (UZA), Antwerp, Belgium; ^4^ Department of Family Medicine and Population Health, University of Antwerp, Antwerp, Belgium; ^5^ Department of Public Health and Primary Care, Ghent University, Ghent, Belgium; ^6^ Department of Public Health and Primary Care, KU Leuven, Leuven, Belgium; ^7^ Department of Patient Care, Antwerp University Hospital (UZA), Antwerp, Belgium

**Keywords:** asthma, adolescent, young adult, transition, respiratory care

## Abstract

**Introduction:**

Asthma is a chronic condition that affects millions of adolescents and young adults (AYA) worldwide. The transition from pediatric to adult care presents unique challenges for this population, affecting their self-management, quality of life and overall health outcomes. This systematic review aims to consolidate the available evidence on challenges encountered by AYA with asthma during the transition period from child to AYA and on the key elements of transitional care for AYAs with asthma including the outcomes achieved, ultimately enhancing outcomes.

**Methodology:**

A systematic literature search was performed in PubMed, Embase, Medline, Scopus, and Web of Science from their inception to October 2, 2023, to provide an overview of currently available literature. Primary quantitative and qualitative studies, published in peer-reviewed journals that focused on AYA with a confirmed diagnosis of asthma were considered if they focused on challenges encountered by AYA with asthma during the transition process and/or components of transitional care and their outcomes assessed.

**Results:**

A total of 855 studies were initially identified and 6 articles were included in this systematic literature review. Several challenges experienced by AYA with asthma were identified including maintaining medication adherence, the need to take responsibility and being involved, understanding their condition and its severity, feeling left out of the care system, and experiencing a lack of engagement. The identified transitional care components included a standardized form for medical data transmission, a joint consultation and to offer several longer consultations.

**Conclusion:**

Several international guidelines for asthma care recommend implementing transition programs in the care for AYA with asthma. Such transition programs should include a comprehensive and individualized approach addressing several challenges faced, to ensure optimal outcomes post-transition. However, to date, data on effective components of transitional care facilitating good outcomes were found to be limited. This systematic review underscores the need for larger studies evaluating the effect of the components of transition programs.

## 1 Introduction

Worldwide, asthma is the most frequently diagnosed chronic respiratory disease in children (1–3). According to the Global Burden of Disease Report 2019, about 22 million (95% UI 15–31 million) children were estimated to be affected (4). The number of incident asthma cases in individuals aged 15–19 years was close to 2.4 million (4) with a prevalence of 10.8% (95% CI; 8.56–13.51) (5). For individuals aged 20–24 years, the prevalence of asthma was 8.7% (95% CI: 6.83–10.91) (5). Asthma is an inflammatory disorder of the airways associated with bronchial hyper-responsiveness, reversible restriction of airflow and symptoms like dyspnea, cough, wheezing and chest tightness (1, 6–8). The healthcare needs of patients with asthma vary throughout time and in intensity. While asthma remains a non-curable disease, good management can control it and enable patients to live full and active lives (1, 9). The pharmacological treatment of asthma patients needs to be regularly evaluated. In patients with partially controlled or uncontrolled asthma, emergency department visits or hospitalizations may occur due to asthma exacerbations (10–13).

Over the past few decades, advancements in innovative treatments have significantly improved the survival prospects of adolescents living with chronic diseases, offering a reasonable expectation that many of them will reach adulthood (14–17). However, as they transition into adolescence, these patients require lifelong specialized care tailored to their age-specific needs. During this critical phase, there is an anticipation that these adolescents will shift from child-oriented care to adult-focused care settings to guarantee the continuity of their care (14–17).

This shift from child-oriented to adult-focused healthcare poses significant challenges for adolescents and young adults (AYA) grappling with chronic illnesses (14). These individuals, along with their parents and healthcare providers, have identified numerous obstacles during this transitional phase, such as altered therapy-adherence, health-related quality of life, self-management, supportive relationships and psychological factors (12, 14–21). Alarmingly, outcomes tend to deteriorate as AYA transition from pediatric care to adult care (14). As many as 40% of adolescents experience a loss of access to specialized care during the transition from pediatric to adult care (14). Despite ongoing efforts, guidelines aimed at addressing these transitional care difficulties are mostly eminence-based instead of evidence-based, still making it crucial to establish best practices in this realm (12, 22). The available evidence is small scaled, often addressing specific conditions and focusing on one aspect of transitional care such as patient education, improving therapy adherence, or supporting self-management (12, 22–28).

Transfer of care can be defined as “*an event or series of events through which adolescents with a chronic condition move their care from a pediatric to an adult-oriented healthcare environment*” (28). Such a transfer of care includes a relocation of care from a pediatric to an adult-oriented setting to guarantee that patients receive continuous care in alignment with their specific healthcare needs as adults (28). Preceding the actual transfer of care is a period called transition. Transition can be defined as “*the change from one stage of life, or physical or mental condition or from one social role to another, which temporarily disrupts normal life and requires a period of adjustment*” (28). Hence, to avoid an abrupt and unprepared transfer of care, patients should receive guidance and support during this transition period, enabling them to gradually adapt to the set of responsibilities, tasks and expectations encountered in adult life and healthcare (28).

According to the current set of recommendations and guidelines, such as the GINA annual report 2023 (12) and the EAACI guidelines 2020 (22), there is a need for the establishment and implementation of transition programs for AYA with asthma. To date, these recommendations, however, do not give any details on the building blocks, content, or key elements of such a transition program. The transitional care needs may differ greatly among AYA with asthma depending on the severity of their disease and its management status (12, 22, 27). To facilitate the establishment of evidence-based transition programs for AYA with asthma, a critical appraisal and synthesis of the current body of evidence will help to identify key elements found to be effective in a transition program, specifically for AYA with asthma.

The objective of this systematic literature review is to evaluate and consolidate (i) the available evidence on challenges encountered by AYA with asthma during the transition period from child to AYA and (ii) on the key elements of transitional care for AYAs with asthma including the outcomes achieved, ultimately enhancing the transition process.

## 2 Methods

### 2.1 Search strategy

To identify eligible studies, an extensive systematic search was performed in PubMed, Embase, Medline, Scopus, and Web of Science from their inception to October 2, 2023. The search strings applied in the respective databases are detailed in Table 1. To extend the initial search, the snowball technique was applied. This involved reviewing the reference lists of pertinent publications to find other possible relevant publications. The review and reporting are performed in line with the Preferred Reporting Items for Systematic Reviews and Meta-Analysis statement (PRISMA) (29).

**Table 1:** Search strings for each database.

PubMed	(“Asthma"[MeSH Terms] OR Asthma*[Title/Abstract] OR “Anti-asthmatic agents"[MeSH Terms] OR “Bronchial Hyperreactivity"[MeSH Terms] OR “Respiratory Hypersensitivity"[MeSH Terms] OR Reactive airway*[Title/Abstract] OR Anti-asthmatic*[Title/Abstract] OR Antiasthmatic*[Title/Abstract] OR “Bronchial Hyperreactivit*"[Title/Abstract] OR “Respiratory Hypersensitivit*"[Title/Abstract] OR “respiratory tract allerg*"[Title/Abstract] OR “Airway Hyper-Responsiveness"[Title/Abstract] OR “Airway Hyperresponsiveness"[Title/Abstract]) AND (“transitional care"[MeSH Terms] OR “Transition to Adult Care"[Mesh] OR “transitional care*"[Title/Abstract] OR “transition program*"[Title/Abstract] OR “transfer of care*"[Title/Abstract] OR “transfer to adult care*"[Title/Abstract] OR “transition of care*"[Title/Abstract] OR “healthcare transition*"[Title/Abstract] OR “transition care*"[Title/Abstract] OR “transition to adulthood"[Title/Abstract] OR “transition readiness"[Title/Abstract] OR “health-care transition*"[Title/Abstract] OR “transition from pediatric*"[Title/Abstract] OR transition from adolescence[Title/Abstract] OR “transition from paediatric*"[Title/Abstract] OR “transition to adult"[Title/Abstract] OR “transfer from Pediatric to Adult Care*"[Title/Abstract] OR “transferring to Adult Care*"[Title/Abstract] OR “transition model*"[Title/Abstract] OR “transition theor*"[Title/Abstract])
Embase	(‘asthma'/exp OR ‘antiasthmatic agent'/exp OR ‘bronchus hyperreactivity'/exp OR ‘respiratory tract allergy'/exp OR (Asthma* OR Anti-asthmatic* OR antiasthmatic* OR ‘Bronchial hyperreactivit*’ OR ‘bronchus hyperreactivit*’ OR ‘Respiratory Hypersensitivit*’ OR ‘respiratory tract allerg*’ OR ‘Reactive airway*’ OR ‘Airway Hyper-Responsiveness’ OR ‘Airway Hyperresponsiveness’):ti,ab,kw) AND (‘transitional care'/exp OR ‘transition to adult care'/exp OR (‘transitional care*’ OR ‘transition program*’ OR ‘transfer of care*’ OR ‘transfer to adult care*’ OR ‘transition of care*’ OR ‘healthcare transition*’ OR ‘transition care*’ OR ‘transition to adulthood’ OR ‘transition readiness’ OR ‘health-care transition*’ OR ‘transition from pediatric*’ OR ‘transition from adolescence’ OR ‘transition from paediatric*’ OR ‘transition to adult’ OR ‘transfer from Pediatric to Adult Care*’ OR ‘transferring to Adult Care*’ OR ‘transition model*’ OR ‘transition theor*’):ti,ab,kw)
Medline	(Exp Asthma/ OR Asthma*.ti,ab,kw OR Exp Anti-asthmatic agents/ OR Exp Bronchial Hyperreactivity/ OR Bronchial hyperreactivit*.ti,ab,kw OR bronchus hyperreactivit*.ti,ab,kw OR Exp Respiratory Hypersensitivity/ OR Respiratory Hypersensitivit*.ti,ab,kw OR respiratory tract allerg*.ti,ab,kw OR Reactive airway*.ti,ab,kw OR Anti-asthmatic*.ti,ab,kw OR antiasthmatic*.ti,ab,kw OR Airway Hyper-Responsiveness.ti,ab,kw OR Airway Hyperresponsiveness.ti,ab,kw) AND (Exp transitional care/ OR transitional care*.ti,ab,kw OR Exp Transition to Adult Care/ OR transition to adult care.ti,ab,kw OR transition program*.ti,ab,kw OR transfer of care*.ti,ab,kw OR transfer to adult care*.ti,ab,kw OR transition of care*.ti,ab,kw OR healthcare transition*.ti,ab,kw OR transition care*.ti,ab,kw OR transition to adulthood.ti,ab,kw OR transition readiness.ti,ab,kw OR health-care transition*.ti,ab,kw OR transition from pediatric*.ti,ab,kw OR transition from adolescence.ti,ab,kw OR transition from paediatric*.ti,ab,kw OR transition to adult.ti,ab,kw OR transfer from Pediatric to Adult Care*.ti,ab,kw OR transferring to Adult Care*.ti,ab,kw OR transition model*.ti,ab,kw OR transition theor*.ti,ab,kw)
Scopus	TITLE-ABS-KEY((Asthma* OR “Reactive airway” OR Anti-asthmatic* OR Antiasthmatic* OR “Bronchial Hyperreactivit*” OR “bronchus hyperreactivit*” OR “Respiratory Hypersensitivit*” OR “respiratory tract allerg*” OR “Airway Hyper-Responsiveness” OR “Airway Hyperresponsiveness”) AND (“transitional care*” OR “transition program*” OR “transfer of care*” OR “transfer to adult care*” OR “transition of care*” OR “healthcare transition*” OR “transition care*” OR “transition to adulthood” OR “transition readiness” OR “health-care transition*” OR “transition from pediatric*” OR transition from adolescence OR “transition from paediatric*” OR “transition to adult” OR “transfer from Pediatric to Adult Care*” OR “transferring to Adult Care*” OR “transition model*” OR “transition theor*”))
Web of Science	TS = (Asthma* OR “Reactive airway” OR Anti-asthmatic* OR Antiasthmatic* OR “Bronchial Hyperreactivit*” OR “bronchus hyperreactivit*” OR “Respiratory Hypersensitivit*” OR “respiratory tract allerg*” OR “Airway Hyper-Responsiveness” OR “Airway Hyperresponsiveness”) AND (“transitional care*” OR “transition program*” OR “transfer of care*” OR “transfer to adult care*” OR “transition of care*” OR “healthcare transition*” OR “transition care*” OR “transition to adulthood” OR “transition readiness” OR “health-care transition*” OR “transition from pediatric*” OR transition from adolescence OR “transition from paediatric*” OR “transition to adult” OR “transfer from Pediatric to Adult Care*” OR “transferring to Adult Care*” OR “transition model*” OR “transition theor*”)

### 2.2 Selection criteria

Primary quantitative and qualitative studies, published in peer-reviewed journals, were considered. Studies were required to focus on adolescents and young adults with a confirmed diagnosis of asthma and needed to focus on challenges encountered during the transition process and/or components of transitional care for AYAs with asthma and their outcomes. Adolescents encompassed individuals within the age range of 10–19 years and young adults within the range of 19–25 years, as defined by the World Health Organization (WHO) (30).

Studies were excluded when the outcomes of transition were not specifically described. Additionally, opinion papers, editorials, letter to the editor or case studies were excluded. Papers which were not written in English were excluded.

### 2.3 Study selection

**Figure 1:**
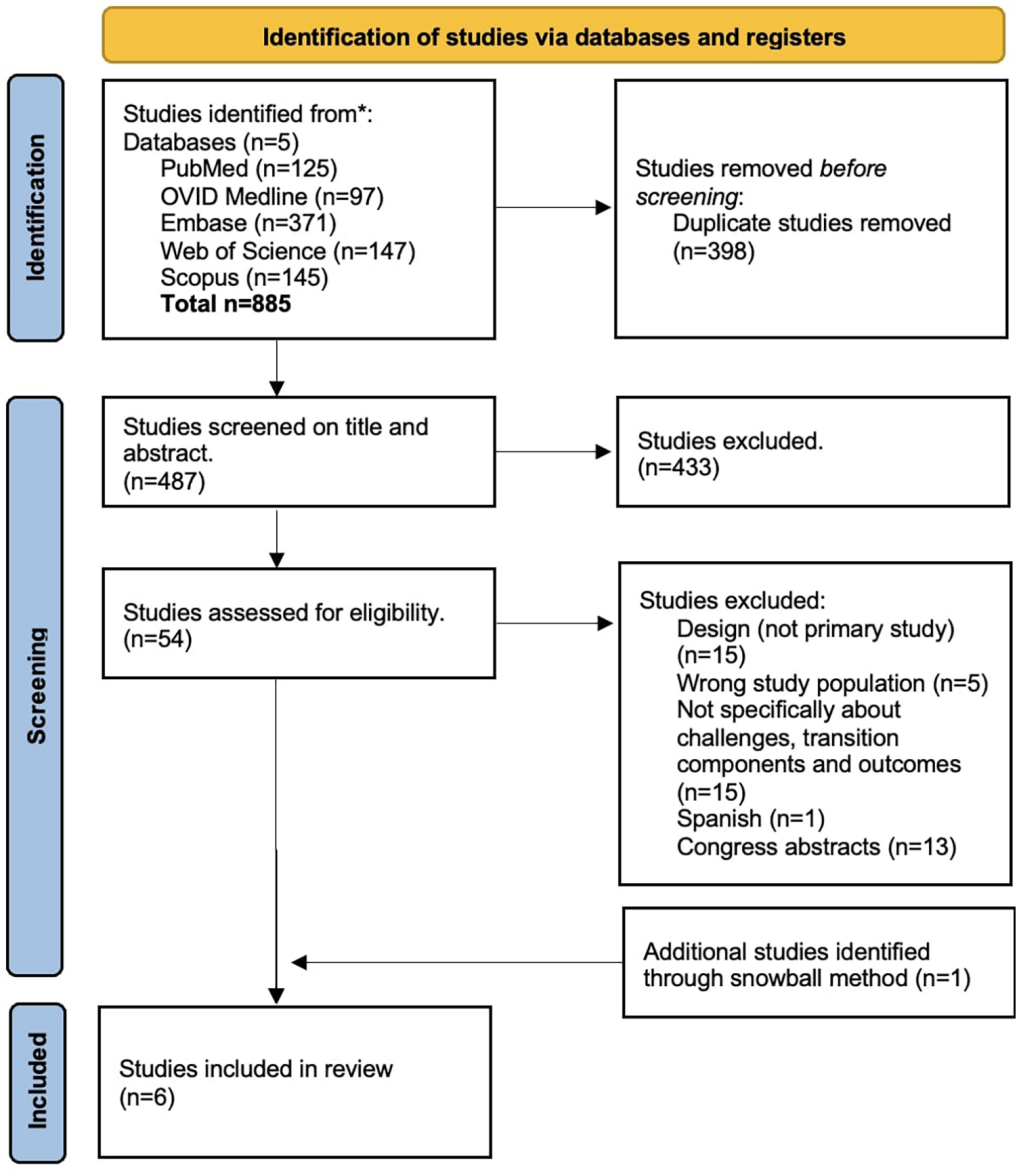
PRISMA flowchart.

The PRISMA flowchart is shown in Figure 1. The search yielded 885 records. After completing the initial search, snowballing, and eliminating 398 duplicates, the next step involved two investigators (LA, KVH) independently screening 487 titles and abstracts for eligibility. A total of 397 references were deemed not in line with the research question. Of these 54 remaining articles, 13 reports were not retrieved in full text because they were congress abstracts and the full text was not yet available. Subsequently, a dual review of 41 full-text articles was carried out to assess eligibility based on the set of selection criteria (LA, YVH or NJ). In cases where discrepancies arose, these were resolved through consensus meetings between the investigators. A total of 35 publications were excluded based on full-text assessment (reasons mentioned in flowchart). Finally, six articles were included in this systematic review.

### 2.4 Data extraction

Data were systematically extracted from included articles by one researcher (LA) comprising author, year of publication, country, study characteristics (i.e., setting, study design, duration, data collection, methodology, and sample size), challenges experienced by AYA during the transition period, transition program components designed for AYA with asthma and the outcomes assessed.

### 2.5 Outcomes

The challenges faced by AYA with asthma experienced during the transition process were examined through the multifaceted nature of these challenges, encompassing emotional, cognitive, and healthcare-related aspects. For the qualitative studies the challenges of AYA were described as themes.

Any transitional care components designed to enhance the transitional care for AYAs moving towards adulthood and adult-oriented health services were examined. These components encompassed a range of approaches, such as dedicated adolescent units, joint consultations, and the utilization of specialized key workers. Studies were included with transitional care components or programs regardless of the duration of the interventions or the specific timing of the interventions.

Any disease-specific patient outcome(s), assessed using validated measures were identified, including asthma control using the Asthma Control Test (ACT), lung function, bronchial challenge with histamine, exercise test and BMI, post-transfer mortality, number of hospital, ICU and/or ED admissions.

### 2.6 Critical appraisal

The identified set of articles involved three cohort studies, one quasi-experimental study, and two qualitative studies. The Newcastle-Ottawa Scale (NOS) for assessing the quality of non-randomized studies and the Critical Appraisal Skills Programme (CASP) for Qualitative research were used to assess the methodological quality and the risk of bias in the selected studies (31, 32). The critical appraisal was performed by two researchers independently (LA, NJ or YVH). The NOS uses an 8-point classification system, while CASP for qualitative research consists of 10 questions to score with a “yes”, “no” or “can't tell”.

### 2.7 Data analysis

The studies' clinical heterogeneity was assessed by considering their varying clinical characteristics, such as the population, intervention methods, outcome evaluation, and follow-up duration. The studies were presented in a narrative format. Descriptive statistics were described and are presented as means ± standard deviations (SD) if data were normally distributed, and medians with interquartile range (IQR) if data were not normally distributed. Nominal variables were described using absolute numbers (*n*) and percentages (%). Data retrieved from the qualitative studies was analyzed using thematic analysis.

## 3 Results

### 3.1 Characteristics of the included studies

The PRISMA flowchart is shown in Figure 1. A total of six studies were included in this systematic literature review. Characteristics of the included studies are presented in Tables 2, 3. Publication years ranged from 2009 to 2022. Three studies were performed in Sweden (33–35), one in France (36), one in Canada (37) and one in the USA (38). The studies of Bergström et al., Cohen et al. and Dufrois et al. described components of a transition program and investigated the impact on a set of clinical outcomes in AYA with asthma; while the other three studies described the challenges experienced by AYA with asthma during the transition period (35–38). Three studies are cohort studies (33, 34, 37), one study is a quasi-experimental study (36) and two are qualitative studies (35, 38). The age range of included patients varied from 14 to 27 years (33–38). All studies describe patients followed in pediatric hospitals or specialized centers (33–38).

**Table 2:** Characteristics of the included studies about challenges of AYA with asthma.

Author, year	Country	Design	Sample size for analysis	Age range	Challenges for AYA in transition
Ödling et al., 2020 (34)	Sweden	Qualitative study	16	16–24	4 categories: “I have to take responsibility”, “A need of being involved”, “Feeling left out of the system”, and “Lack of engagement”.
Ödling et al., 2020 (35)	Sweden	Cohort study	1808	16–24	Medication adherence
Zaeh et al., 2022 (38)	USA	Qualitative study	14	14–27	5 categories: adherence to medication, taking responsibility for asthma self-management, understanding asthma condition and severity, embarrassment about medication use, lifestyle

**Table 3:** Characteristics of included studies about transitional components and their outcomes.

Author, year	Country	Design	Sample size for analysis	Age range	Transition components	Outcomes of transitional components
Bergström et al., 2010 (33)	Sweden	Cohort study	150	16, 2–21, 8	Adult specialized care and primary care setting	Pulmonary function rarely deteriorated. Bronchial hyperresponsiveness persisted. Negative impactors: female, poor adherence to treatment, inhalation of steroids Mild/moderate asthma managed effectively equally with primary or specialized care
Cohen et al., 2016 (37)	Canada	Cohort study	104, 497 Asthma 92,254	16–20	Transition but components not specified	Stable patterns of mortality, overall healthcare use after transfer Preventative visits declined; specialized visit increased. ED use increased slightly.
Dufrois et al., 2022 (36)	France	Quasi- experimental study	54	18	Transition center with joint consultations, a standardized form for transmission of medical data and longer consultations if needed	13 patients (25%) were lost to follow-up after an average of 22.4 months of follow-up. Three-quarters (73%) of patients had well controlled asthma with an ACT score ≥ 20 during transition and the majority were able to maintain good control and respiratory function (>60% FEV1 > 80%) during follow-up in adult pulmonology. Among the patients that answered the questionnaire, 64.8% were satisfied with the transition process but several improvements can be proposed, including early discussion of medical plan, implementation of procedures to reduce loss of follow-up

### 3.2 Quality and publication bias

Based on the NOS, the overall quality of the four non-randomized studies was moderate to high (31). The results can be found in Table 4. Three out of four studies obtained a maximum score of six (33, 34, 36). One study did not describe anything about the adequacy of follow-up and therefore, scored five out of six on overall quality (37).

**Table 4:** Newcastle-Ottowa scale (NOS) for assessing the quality of nonrandomized studies in meta-analyses.

	Bergström et al. (33)	Cohen et al. (37)	Dufrois et al. (36)	Ödling et al. (34)
Selection				
Representativeness of the exposed cohort	*	*	*	*
Selection of the non-exposed cohort	NR	NR	NR	NR
Ascertainment of exposure	*	*	*	*
Outcome not present at start of study	*	*	*	*
Comparability				
Comparability of cohorts on the basis of the design or analysis	NR	NR	NR	NR
Outcome				
Assessment of outcome	*	*	*	*
Long enough follow-up for outcome to occur	*	*	*	*
Adequacy of follow-up	*	-	*	*
Total Quality score	6/6	5/6	6/6	6/6

NR, not relevant.

Based on the CASP, both qualitative studies obtained a high score of nine out of ten of overall quality (32). The results can be found in Table 5. One study did not fulfill the criterion of sufficiently rigorous data analysis because no data saturation was mentioned, and the authors gave very shallow descriptions of the performed analysis (35). The other study did not fulfill the criterion of data collection addressing the research issue because participants with low digital skills were excluded and did not mention data saturation (38).

**Table 5:** Critical appraisal skills programme (CASP) for qualitative research.

	Ödling et al. (35)	Zaeh et al. (38)
Was there a clear statement of the aims of the research?	Yes	Yes
Is a qualitative methodology appropriate?	Yes	Yes
Was the research design appropriate to address the aims of the research?	Yes	Yes
Was the recruitment strategy appropriate to the aims of the research?	Yes	Yes
Was the data collected in a way that addressed the research issue?	Yes	No
Has the relationship between researcher and participants been adequately considered?	Yes	Yes
Have ethical issues been taken into consideration?	Yes	Yes
Was the data analysis sufficiently rigorous?	No	Yes
Is there a clear statement of findings?	Yes	Yes
How valuable is the research?	Yes	Yes
Overall quality score	9/10	9/10

### 3.3 Challenges faced by AYA with asthma during transition

The following challenges faced by AYA during transition were identified: medication adherence (34, 38), taking responsibility, the need of becoming involved, understanding their condition and its severity (35, 38), feeling left out of the care system (35), and experiencing a lack of engagement (35).

The studies by Zaeh et al. and Ödling et al. described challenges encountered by AYA with asthma during the transition period in terms of their medication adherence (34, 38). At the follow-up of 16-year-old patients with current asthma, dispensation of Short-Acting Beta Agonists (SABA) (ẋ: 2.3) and any Inhaled Corticosteroids (ICS) (ẋ: 1.1) was found to be low (34). These rates notably declined after reaching 18 years of age across all asthma types (*p* < .01), except for severe asthma (34). In patients with current asthma, a minimum of one dispensation of SABA, before turning 18 years old, was observed in 70% (103 out of 147) as compared to 50% (73 out of 147) after reaching 18 years of age. Before the age of 18 years, about 73% (107 out of 147) had at least one prescription for inhaled corticosteroids (ICS), whereas after the age of 18, this percentage dropped to 50% (74 out of 147) (34). Zaeh et al. outlined various challenges influencing the medication adherence in AYA with asthma. Using a socio-ecological framework, several themes emerged, including individual factors such as taking responsibility for asthma management and understanding the severity of the condition. Interpersonal factors included feelings of embarrassment associated with using asthma medication, while community-related factors included life demands that altered medication adherence (38). This study of Zaeh et al. also identified societal factors such as the cost of asthma medications, challenges with health insurance, school-related factors and difficulty obtaining medication refills (38).

Zaeh et al. and Odling et al. both identified the themes of taking responsibility, the need of becoming involved and understanding their condition and its severity. AYA highlighted they experienced an increased need to take on more responsibility following a transfer to adult care setting (35, 38). They were used to receiving proactive support, including appointment reminders and parental support in the pediatric setting, whereas in the adult care setting they noticed a significant change. They felt the need to mature quickly and take full responsibility for managing their healthcare needs (38). AYA expressed a desire for more direct involvement in their asthma as they moved from childhood to adolescence (35). As information during childhood was mainly directed to their parents, adolescents longed for healthcare providers to actively involve them in their asthma self-management (35, 38). AYA expressed the need to have better comprehension of their disease, such as the nature of asthma, the reasons for taking certain medications, and the consequences of therapy non-adherence (38). In the adult setting, AYA felt the weight of taking full responsibility for their healthcare needs, which included actively requesting care, taking their asthma seriously and maintaining communication with healthcare providers (35). The perceived difference in responsibility was, however, less pronounced when care was provided by the same primary care provider from child- to adult-focused care, even without parental involvement. AYA also expressed the need to gain some level of assertiveness in adult care. They expressed a need to advocate for their healthcare needs towards healthcare providers. The transition marked a significant shift for them, demanding greater maturity, self-reliance, and self-management (35, 38).

Additionally, the study of Odling et al. identified two more themes. AYA reported feeling left out of the care system and experiencing a lack of engagement. They expressed frustration regarding the lack of information about transitioning to adult healthcare, leading to uncertainty about where to go for help, getting prescriptions or understanding next steps in the transition process (35). In cases where the transition was not facilitated, they felt excluded from the healthcare system, experiencing anxiety due to unfamiliarity with adult healthcare providers and felt directionless. In adult healthcare, their visits often felt unproductive, lacking personal interaction, and focusing solely on prescription renewals, with no discussion or evaluation of their treatment (35).

### 3.4 Description of components of transition programs for AYA with asthma

The study of Dufrois et al. described the use of a standardized form for the transmission of medical data by the pediatric pulmonology service. This was conceived jointly by all medical teams involved in the program and consisted of a summary of the patients' medical history, the performed investigations, the treatments received and information on educational attainment, family, and social life. This form was completed during a joint meeting between the pediatric and adult pulmonologist where they discussed the different aspects of disease management before the first consultation or directly by the patient. The adult pulmonologist took charge of organizing the first consultation in the adult center at a time purposed by the pediatric team, with the family having no role to play. All patients were offered several longer consultations preceded by a meeting with a nurse who provided practical information regarding the practicalities at the adult center (36). It was not specified how long the longer consultations were.

The study of Bergström et al. describes a guided transfer of care to specific adult asthma clinics or to primary care (33).

### 3.5 Outcomes achieved after implementation of transitional care components

The following outcomes after implementation of transitional care components were identified: asthma control (33, 36), lung function and bronchial challenge with histamine (33, 36), exercise test and BMI (33), loss of follow-up (33, 36), post-transfer mortality (37), number of hospital (37), ICU (37) and ED admissions (37).

Dufrois et al. described that most patients had well controlled asthma at the time of transfer, their mean Asthma Control Test (ACT) score was 19 ± 6 (median: 21) and maintained good asthma control and good respiratory function after transfer. The continuation of follow-up in an adult center contributed to the maintenance of well controlled disease and good respiratory function at 3 years after transition (36). Among the patients with well controlled asthma at the time of transition, 40% maintained an ACT score of ≥20 for the entire follow-up, up to 3 years after transition. However, 3/12 patients with uncontrolled asthma at transition did not have any improvement in their asthma control and maintained an ACT score of ≤15 3 years after transition (36).

Bergström et al. reported a mean FEV1 at the time of transition of 95.5% that increased significantly at 5 years after transition (32). In the study of Dufrois et al. the proportion of patients with FEV1 > 80%, 60%–80% and <60% remained stable (36).

In the study of Bergström et al. an exercise test was performed pre-transition and after five years, the mean total oxygen uptake decreased from 3.1 L/min (SD ± 0.9) to 2.9 L/min (SD ± 0.8; *p* < .047) and in relation to weight, from 48.3 (SD ± 10.6) to 40.6 ml/kg/min (SD ± 8.9; *p* < 0.001) between these two time-points. Engagement in regular exercise outside of school or work, exceeding 2 h per week, decreased from 28% upon entry to 16% at the five-year follow-up (33). Most asthmatic adolescents transitioned into adulthood with bronchial hyper-responsiveness, notably prevalent in individuals with a positive skin prick test (*p* < .003), among females (*p* < .01), and in correlation with inadequate adherence to asthma treatment (*p* < .002). These specific subgroups face a higher risk of developing chronic asthma and/or experiencing progressively severe symptoms during transition (33). The study of Bergström et al. also described that in terms of bronchial responsiveness there is no significant difference for individuals with mild to moderate asthma to seek care at a specialized asthma clinic over receiving primary care (OR 0.61; 95% CI: 0.26–1.44; *p* = 0.26) (33).

In the studies of Bergström et al. and Dufrois et al. the loss of follow-up changed from, respectively, 30.6%–36.5% after their transition to the adult center (33, 36).

The study of Cohen et al. found that there was no significant difference in post-transfer mortality rates in the asthma cohort with *p* = .05 (pre-transfer 0.1% vs. post-transfer 0.1%). The proportion of patients with a hospital admission slightly increased (5.8% vs. 6.3%; *p* < 0.001), while the number of hospital ICU admissions did not significantly change (0.5% vs. 0.5%; *p* = .60). The number of ED visits did, however, increase (44.5% vs. 45.9%; *p* < .001) (37). However, Dufrois et al. reported fewer ED visits and hospital admissions for AYA with asthma three years after transition (19.2% vs. 13.5%) (36).

## 4 Discussion

The transfer from pediatric to adult care poses several challenges for AYA with asthma. Based on the current body of evidence, safeguarding medication adherence during the transition to adulthood appears to be a major challenge during the transition to adulthood. A decline in dispensation of asthma medications, such as SABA and ICS, after reaching 18 years of age, was observed (36). Various factors appear to influence medication adherence in AYA with asthma, including individual, interpersonal, community-related, and societal factors (35). Furthermore, AYA express a need for a better understanding of their disease, increased responsibility, and assertiveness in adult care settings. Additionally, AYA report feeling left out of the healthcare system and a lack of engagement, leading to uncertainty and anxiety (35). These challenges illustrate a need for the establishment of patient-centered transition programs tailored to the needs of AYA with asthma.

After the implementation of a transitional care component, there was variability in outcomes among AYA with asthma as well as the outcomes measured after transition. While some patients maintained well-controlled asthma and good respiratory function, others experienced challenges in asthma control and maintenance of their respiratory function (33, 36). This highlights the importance of preparation, support and information transfer between medical teams for a successful transition (36). The GINA guidelines and the EAACI guidelines provide recommendations for the transition of AYA with allergy and asthma, but do not address the specific challenges faced by AYA with asthma (12, 22). The study by Dufrois et al. emphasized the importance of early discussion of the medical plan and the implementation of procedures to reduce loss to follow-up, indicating the need for continuous support and monitoring post-transition (33, 36).

Many healthcare systems struggle to provide targeted interventions that address the unique needs and concerns of AYA with asthma during this transition phase. The lack of data supporting the development and implementation of evidence-based transition programs for AYA with asthma leads to potential gaps in follow-up care and increased risks of suboptimal disease management. It impacts the ability of healthcare providers to offer tailored support, education, and resources necessary for AYA to effectively manage their asthma as they transition into adulthood. To date, as identified by this systematic review, there is very little literature published specifically examining the transition process in AYA with asthma (33). This remains surprising as asthma is the most common chronic medical condition in children (2) and highlights the need for further research to determine optimal care for AYA with asthma during transition.

Hence, the challenges faced by AYA with asthma during transition are multifaceted, encompassing challenges to maintain medication adherence, adapting to increasing responsibility, growing engagement, and dealing with anxiety and uncertainty. Person-tailored transition programs are essential to address these challenges and ensure the continuity of care for AYA with asthma as they move to adulthood and adult-focused care settings.

The findings of this systematic review should, however, be interpreted in light of some methodological considerations. The strength of this review is the performance of a thorough literature search. The selection of studies for inclusion, along with the extraction and analysis of data, adhered to a strict and systematic approach in which several researchers independently participated. The selected set of studies was found to have sound methological quality in line with their study design, using validated tools for the performance of a critical appraisal (31, 32). The data extraction in this systematic review was performed by one researcher which means there is a risk of bias.

Concerns could be raised related to inconsistencies between study results due to the heterogeneous and complex nature of the interventions and variations in outcome measures in the selected papers. This review summarizes a heterogeneous group of studies; therefore, conclusions should be critically approached. Studies lacked comprehensive descriptions of their transition procedures. This absence of detailed information poses challenges in categorizing similar transition interventions and renders it impossible to correlate outcomes with a particular transition process. It is important to note that the timing and methods of transition can differ between countries. Most adolescents with chronic illnesses typically begin transitioning to adult health services as they complete their secondary education (14, 15). The method of transition or the content of a transition program varies greatly because there is no guideline or standardized protocol for the transition of AYA with asthma.

It is important to note that the study of Bergström et al. was conducted in a specific setting in Sweden, therefore the results might not by generalized to other settings (33). The study of Odling et al. formulates medication adherence as a challenge for AYA with asthma (34). There were no components of transition specified so it could not be considered an outcome achieved by a transition program.

A variety of outcomes were assessed after implementation of a transition program, including, asthma control (33, 36), lung function and bronchial challenge with histamine (33, 36), exercise test and BMI (33), loss of follow-up (33, 36), post-transfer mortality (37), number of hospital (37), ICU (37) and ED admissions (37). However, to the best of our knowledge, no studies assessing the impact of a transition program on for example, access to specialized care, reduction in transition barriers, experience of care in AYA with asthma, are currently performed. Transition to adult care is a complex intervention involving numerous steps, each with the potential, individually or collectively, to impact outcomes. Large-scale (quasi-) experimental trials with sound methodological quality are important to specify which transition components are found to be effective in achieving favorable patient-related outcomes of the transition process of AYA with asthma.

## 5 Conclusion

In this systematic review, only six studies were identified investigating challenges faced by AYA with asthma during the transitional period, transitional components, and their respective outcomes. Several challenges where identified, including safeguarding medication adherence, increasing responsibility, understanding their condition and its severity, feeling left out of the care system, and experiencing a lack of engagement. The transitional care components identified included, a standardized form for the transmission of medical data, a joint consultation and offering multiple longer duration consultations.

This systematic review underscores the need for larger studies evaluating the effect of the components of transition programs to achieve beneficial outcomes for AYA with asthma. Future studies should delve deeper into the specific transitional care components and given the identified challenges, it is important to conduct research aimed at tailoring transition programs specifically to the needs of AYA with asthma. To assess the long-term effectiveness of transitional care, longitudinal studies are warranted.

## Data Availability

The original contributions presented in the study are included in the article/Supplementary Material, further inquiries can be directed to the corresponding author.

## References

[1] McCoy EM Kink RJ Harrold PL Longjohn MK Meredith ML Pishko SD. Implementing a standardized clinical pathway leads to reduced asthma admissions and health care costs. Pediatr Qual Saf. (2018) 3(4):e091. 10.1097/pq9.0000000000000091 30229202 PMC6135551

[2] Martin MA Press VG Nyenhuis SM Krishnan JA Erwin K Mosnaim G Care transition interventions for children with asthma in the emergency department. J Allergy Clin Immunol. (2016) 138(6):1518–25. 10.1016/j.jaci.2016.10.012 27931533 PMC5327498

[3] Sneller H Keenan K Hoppa E. A quality improvement initiative to improve the administration of systemic corticosteroids in the pediatric emergency department. Pediatr Qual Saf. (2020) 5(3):e308. 10.1097/pq9.0000000000000308 32656471 PMC7297401

[4] Zhang D Zheng J. The burden of childhood asthma by age group, 1990–2019: a systematic analysis of global burden of disease 2019 data. Front Pediatr. (2022) 10:823399. 10.3389/fped.2022.823399 35252064 PMC8888872

[5] Song P Adeloye D Salim H Dos Santos JP Campbell H Sheikh A Global, regional, and national prevalence of asthma in 2019: a systematic analysis and modelling study. J Glob Health. (2022) 12:04052. 10.7189/jogh.12.04052 35765786 PMC9239324

[6] Ardura-Garcia C Stolbrink M Zaidi S Cooper PJ Blakey JD. Predictors of repeated acute hospital attendance for asthma in children: a systematic review and meta-analysis. Pediatr Pulmonol. (2018) 53(9):1179–92. 10.1002/ppul.24068 29870146 PMC6175073

[7] Orellano P Quaranta N Reynoso J Balbi B Vasquez J. Effect of outdoor air pollution on asthma exacerbations in children and adults: systematic review and multilevel meta-analysis. PLoS One. (2017) 12(3):e0174050. 10.1371/journal.pone.0174050 28319180 PMC5358780

[8] Axelsson I Naumburg E Prietsch SO Zhang L. Inhaled corticosteroids in children with persistent asthma: effects of different drugs and delivery devices on growth. Cochrane Database Syst Rev. (2019) 6(6):CD010126. 10.1002/14651858.CD010126.pub2 31194879 PMC6564081

[9] World Health Organization. Asthma (2022). Available online at: https://www.who.int/news-room/fact-sheets/detail/asthma (accessed September 3, 2023).

[10] Global, Health, Metrics. Asthma—level 3 cause. Lancet. (2020) 396(10258):1204–22. 10.1016/S0140-6736(20)30925-9

[11] Volksgezondheid, en, zorg. Astma cijfers en contect, de huidige situatie (2023). Available online at: https://www.vzinfo.nl/astma (accessed September 18, 2023).

[12] Global, initiative, for, asthma. (2023). *Global strategy for asthma management and prevention Main Report*. Available online at: https://ginasthma.org/wp-content/uploads/2023/07/GINA-2023-Full-report-23_07_06-WMS.pdf (accessed September 29, 2023).

[13] Achakulwisut P Brauer M Hystad P Anenberg SC. Global, national, and urban burdens of paediatric asthma incidence attributable to ambient NO(2) pollution: estimates from global datasets. Lancet Planet Health. (2019) 3(4):e166–e78. 10.1016/S2542-5196(19)30046-4 30981709

[14] Pape L Ernst G. Health care transition from pediatric to adult care: an evidence-based guideline. Eur J Pediatr. (2022) 181(5):1951–8. 10.1007/s00431-022-04385-z 35084548 PMC9056438

[15] Schidlow DV Fiel SB. Life beyond pediatrics. Transition of chronically ill adolescents from pediatric to adult health care systems. Med Clin North Am. (1990) 74(5):1113–20. 10.1016/s0025-7125(16)30505-3 2201847

[16] Blum RW Garell D Hodgman CH Jorissen TW Okinow NA Orr DP Transition from child-centered to adult health-care systems for adolescents with chronic conditions. A position paper of the society for adolescent medicine. J Adolesc Health. (1993) 14(7):570–6. 10.1016/1054-139x(93)90143-d 8312295

[17] Rosen DS Blum RW Britto M Sawyer SM Siegel DM, Society for Adolescent Medicine. Transition to adult health care for adolescents and young adults with chronic conditions: position paper of the society for adolescent medicine. J Adolesc Health. (2003) 33(4):309–11. 10.1016/s1054-139x(03)00208-8 14519573

[18] Haahtela T Tuomisto LE Pietinalho A Klaukka T Erhola M Kaila M A 10 year asthma programme in Finland: major change for the better. Thorax. (2006) 61:663–70. 10.1136/thx.2005.055699 16877690 PMC2104683

[19] Ross KR Gupta R DeBoer MD Zein J Phillips BR Mauger DT Severe asthma during childhood and adolescence: a longitudinal study. J Allergy Clin Immunol. (2020) 145:140–6. 10.1016/j.jaci.2019.09.030 31622688

[20] Hovland V Riiser A Mowinckel P Carlsen KH Lødrup Carlsen KC. Early risk factors for pubertal asthma. Clin Exp Allergy. (2015) 45:164–76. 10.1111/cea.12409 25220447

[21] Robinson PD Jayasuriya G Haggie S Uluer AZ Gaffin JM Fleming L. Issues affecting young people with asthma through the transition period to adult care. Paediatr Respir Rev. (2022) 41:30–9. 10.1016/j.prrv.2021.09.005 34686436

[22] Roberts G Vazquez-Ortiz M Knibb R Khaleva E Alviani C Angier E EAACI guidelines on the effective transition of adolescents and young adults with allergy and asthma. Allergy. (2020) 75(11):2734–52. 10.1111/all.14459 32558994

[23] Khaleva E Vazquez-Ortiz M Comberiati P DunnGalvin A Pite H Blumchen K Current transition management of adolescents and young adults with allergy and asthma: a European survey. Clin Transl Allergy. (2020) 10:40. 10.1186/s13601-020-00340-z 33042515 PMC7542112

[24] Axelsson M Emilsson M Brink E Lundgren J Toren K Lotvall J. Personality, adherence, asthma control and health-related quality of life in young adult asthmatics. Respir Med. (2009) 103(7):1033–40. 10.1016/j.rmed.2009.01.013 19217764

[25] Rhee H Wicks MN Dolgoff JS Love TM Harrington D. Cognitive factors predict medication adherence and asthma control in urban adolescents with asthma. Patient Prefer Adherence. (2018) 12:929–37. 10.2147/PPA.S162925 29872278 PMC5973469

[26] Holley S Walker D Knibb R Latter S Liossi C Mitchell F Barriers and facilitators to self-management of asthma in adolescents: an interview study to inform development of a novel intervention. Clin Exp Allergy. (2018) 48(8):944–56. 10.1111/cea.13141 29573024

[27] Sundell K Bergstrom SE Hedlin G Ygge BM Tunsater A. Quality of life in adolescents with asthma, during the transition period from child to adult. Clin Respir J. (2011) 5(4):195–202. 10.1111/j.1752-699X.2010.00218.x 21801321

[28] Knauth A Verstappen A Reiss J Webb GD. Transition and transfer from pediatric to adult care of the young adult with complex congenital heart disease. Cardiol Clin. (2006) 24(4):619–29, vi. 10.1016/j.ccl.2006.08.010 17098515

[29] Moher D Liberati A Tetzlaff J Altman DG The PG. Preferred reporting items for systematic reviews and meta-analyses: the PRISMA statement. PLoS Med. (2009) 6(7):e1000097. 10.1371/journal.pmed.1000097 19621072 PMC2707599

[30] World Health Organization. Adolescent and young adult health (2023). Available online at: https://www.who.int/news-room/fact-sheets/detail/adolescents-health-risks-and-solutions (accessed Oktober 3, 2023).

[31] Wells GA Shea B O’Connell D Peterson J Welch V Losos M Tugwell P. The newcastle-ottawa scale (NOS) for assessing the quality of non- randomised studies in meta-analyses (2009). Available online at: http://www.ohri.ca/programs/clinical_epidemiology/oxford.asp (accessed Oktober 3, 2023).

[32] Critical Appraisal Skills Programme. CASP Qualatative studies Checklist (2023). Available online at: https://casp-uk.net/images/checklist/documents/CASP-Qualitative-Studies-Checklist/CASP-Qualitative-Checklist-2018_fillable_form.pdf (accessed Oktober 3, 2023).

[33] Bergström SE Sundell K Hedlin G. Adolescents with asthma: consequences of transition from paediatric to adult healthcare. Respir Med. (2010) 104(2):180–7. 10.1016/j.rmed.2009.09.021 19889523

[34] Ödling M Andersson N Hallberg J Almqvist C Janson C Bergström A A gap between asthma guidelines and management for adolescents and young adults. J Allergy Clin Immunol Pract. (2020) 8(9):3056–3065.e2. 10.1016/j.jaip.2020.05.034 32522564

[35] Ödling M Jonsson M Janson C Melén E Bergström A Kull I. Lost in the transition from pediatric to adult healthcare? Experiences of young adults with severe asthma. J Asthma. (2020) 57(10):1119–27. 10.1080/02770903.2019.1640726 31328590

[36] Dufrois C Bourgoin-Heck M Lambert N Just J Bregeon A Taillé C Maintenance of asthma control in adolescents with severe asthma after transitioning to a specialist adult centre: a French cohort experience. J Asthma Allergy. (2022) 15:327–40. 10.2147/JAA.S348369 35283635 PMC8909487

[37] Cohen E Gandhi S Toulany A Moore C Fu L Orkin J Health care use during transfer to adult care among youth with chronic conditions. Pediatrics. (2016) 137(3):e20152734. 10.1542/peds.2015-2734 26933203

[38] Zaeh SE Lu MA Blake KV Ruvalcaba E Ayensu-Asiedu C Wise RA “It is kind of like a responsibility thing": transitional challenges in asthma medication adherence among adolescents and young adults. J Asthma. (2022) 59(5):956–66. 10.1080/02770903.2021.1897836 33653199 PMC8458468

